# Crucial Role of Mammalian Glutaredoxin 3 in Cardiac Energy Metabolism in Diet-induced Obese Mice Revealed by Transcriptome Analysis

**DOI:** 10.7150/ijbs.60263

**Published:** 2021-07-13

**Authors:** Ninghui Cheng, Qianxing Mo, Jimmonique Donelson, Lingfei Wang, Ghislain Breton, George G. Rodney, Jin Wang, Kendal D. Hirschi, Xander H. T. Wehrens, Paul A. Nakata

**Affiliations:** 1 USDA/ARS Children Nutrition Research Center, Department of Pediatrics, Baylor College of Medicine, Houston, Texas 77030, USA.; 2 Department of Biostatistics & Bioinformatics, H. Lee Moffitt Cancer Center & Research Institute, Tampa, FL 33612, USA.; 3 Department of Pharmacology and Chemical Biology, Baylor College of Medicine, Houston, TX 77030, USA.; 4 Department of Integrative Biology & Pharmacology, McGovern Medical School, The University of Texas Health Science Center at Houston, Houston, Texas 77030, USA.; 5 Department of Molecular Physiology & Biophysics, Baylor College of Medicine, Houston, TX 77030, USA.; 6 Cardiovascular Research Institute, and Center for Drug Discovery, Baylor College of Medicine, Houston, TX 77030, USA.; 7 Dan L. Duncan Cancer Center, Baylor College of Medicine, Houston, TX 77030, USA.

**Keywords:** Heart failure, glutaredoxin, oxidative stress, cardiac energy metabolism, transcriptome

## Abstract

Obesity is often associated with metabolic dysregulation and oxidative stress with the latter serving as a possible unifying link between obesity and cardiovascular complications. Glutaredoxins (Grxs) comprise one of the major antioxidant systems in the heart. Although Grx3 has been shown to act as an endogenous negative regulator of cardiac hypertrophy and heart failure, its metabolic impact on cardiac function in diet-induced obese (DIO) mice remains largely unknown. In the present study, analysis of Grx3 expression indicated that Grx3 protein levels, but not mRNA levels, were significantly increased in the hearts of DIO mice. Cardiac-specific Grx3 deletion (Grx3 CKO) mice were viable and grew indistinguishably from their littermates after being fed a high fat diet (HFD) for one month, starting at 2 months of age. After being fed with a HFD for 8 months (starting at 2 months of age); however, Grx3 CKO DIO mice displayed left ventricular systolic dysfunction with a significant decrease in ejection fraction and fractional shortening that was associated with heart failure. ROS production was significantly increased in Grx3 CKO DIO cardiomyocytes compared to control cells. Gene expression analysis revealed a significant decline in the level of transcripts corresponding to genes associated with processes such as fatty acid uptake, mitochondrial fatty acid transport and oxidation, and citrate cycle in Grx3 CKO DIO mice compared to DIO controls. In contrast, an increase in the level of transcripts corresponding to genes associated with glucose uptake and utilization were found in Grx3 CKO DIO mice compared to DIO controls. Taken together, these findings indicate that Grx3 may play a critical role in redox balance and as a metabolic switch in cardiomyocytes contributing to the development and progression of heart failure.

## Introduction

Overweight/obesity is an independent risk factor for cardiovascular disease (CVD) and has been associated with adverse effects on cardiac structure, hemodynamics, and function 1. Recent epidemiological studies; however, suggests that heart failure patients with mild obesity have a better prognosis than their leaner counterparts 2. The underlying mechanisms that explain this obesity paradox remain to be fully elucidated. Obesity is often associated with metabolic dysregulation and oxidative stress. Both are considered a unifying link between obesity and cardiovascular complications 3. Experimental studies in rodents indicate that enhancement of antioxidant capacity in the heart plays a protective role in diabetes induced cardiac dysfunction, however, clinical investigations have failed to support the beneficial effect of antioxidants 4. This apparent contradiction may be attributed to the complexity of the regulatory pathways controlling redox balance and cardiac energy metabolism in the heart of obese subjects.

Glutaredoxins (Grxs) are ubiquitous, small heat-stable disulfide oxidoreductases which are one of the major antioxidant systems in the heart 5. Grxs can be categorized into dithiol Grxs, which contain two cysteine residues in their active motifs, and monothiol Grxs which contain a single cysteine residue in their putative motifs 5. Grx3, a monothiol Grx, also termed thioredoxin-like 2 (Txnl2) or protein kinase C interacting cousin of thioredoxin (PICOT), interacts with the protein kinase C theta isoform 6. Previous studies demonstrate that Grx3 is essential for early embryonic growth and development 7, 8. Recent reports revealed that forced expression of *Grx3* in transgenic rat heart could enhance cardiomyocyte contractility by modulating calcineurin-NFAT-mediated signaling and PKCζ activity in the progression of pressure-overload induced heart hypertrophy 9-11. Our recent study indicated, using a cardiac-specific Grx3 deletion mouse model, that Grx3 is an important factor in regulating cardiac hypertrophy and heart failure by modulating both cellular redox homeostasis and Ca^2+^ handling in the heart 12. The metabolic impact of Grx3 on cardiac energy metabolism in diet-induced obese (DIO) mice; however, remains largely unknown.

In this study, we investigated the expression of Grx3 in the heart of DIO mice compared to controls. We characterized the Grx3 CKO DIO mice and showed that disruption of Grx3 in DIO mice led to cardiac dysfunction and heart failure. In addition, we documented the transcriptional regulatory mechanism underlying Grx3 function in cardiomyocytes. Taken together, our findings suggest that the presence of Grx3 is critical for maintaining redox balance and cardiac energy metabolism.

## Materials and Methods

### Reagents

All chemicals were purchased from Sigma-Aldrich (St. Louis, MO, USA) unless stated otherwise. Anti-GAPDH antibody was bought from Chemicon International, Inc. Grx3 monoclonal antibody was made in-house using the full-length human Grx3 recombinant protein. This antibody was validated and used in our previous studies 8, 13, 14.

### Animals

Grx3 cardiac specific knockout (Grx3 CKO) mice were generated as previously described 12. All mouse strains were housed in temperature-controlled environment and fed a standard chow diet (LabDiet 5V5R chow; LabDiet, St. Louis, MO) *ad libitum* unless stated otherwise. Male mice were used in this study and Grx3 floxed mouse (Grx3*^flox/flox^*) littermates were used as controls. Mice were fed with high fat diet (D12492, rodent diet with 60 kcal% fat, Research Diets, Inc., New Brunswick, NJ, USA) for one month starting at the age of 2 months old (until 3 months of age) and for eight months starting at the age of 2 months old (until 10 months of age). All studies were performed according to protocols approved by the Institutional Animal Care and Use Committee of Baylor College of Medicine conforming to the Guide for the Care and Use of Laboratory Animals published by the U.S. National Institutes of Health (NIH Publication No. 85-23, revised 1996).

### Echocardiography

Lightly anesthetized mice (1.5-2.0% isoflurane) were imaged in the left lateral decubitus position with a linear 30-MHz probe (Visualsonic Vevo 2100 Imaging System, Baylor Mouse Phenotyping Core) 12. Digital images were collected at a frame rate of 180 images/s. Two-dimensional images were recorded in parasternal long- and short-axis projections with guided M-mode recordings. All measurements were done blinded to genotypes.

### ROS production assay

Dihydroergotamine (DHE) staining to measure ROS production in cardiomyocytes was done as previously described 12. The DHE fluorescence signals were detected at 610 nm (excitation at 520 nm) using an Olympus Fluoview scanning laser confocal microscopy system. The fluorescent intensity was quantified using Image J.

### NAD^+^ and NADH Assay

20 mg of heart (ventricle) tissues from chow-fed and HFD-fed Grx3 CKO DIO and control mice were used in the measurement of NAD^+^ and NADH using an NAD^+^/NADH assay kit (BioAssay, CA, USA) following the manufacturer's instructions. The NAD^+^/NADH ratio was calculated by dividing the measured amount of NAD^+^ by the amount of NADH.

### Quantitative RT-PCR and microarray analysis

Total RNA was extracted from heart (ventricle) tissues from Grx3 CKO DIO mice and DIO controls either using Trizol reagent (Thermo Fisher Scientific, Waltham, MA) or using the Quick-RNA Miniprep Plus Kit (Zymo Research, Irvine, CA) following the manufacturer's instructions. For q-PCR analysis, purified RNA samples underwent reverse transcription to yield cDNA. qPCR was performed using the SYBR Green-based assay and the Bio-Rad CFX96™ Real-Time PCR Detection System. Differences in gene expression between control and knockout mice were quantified by comparative CT method. Gapdh was selected to serve as the internal control because its expression level was consistent between WT and KO and relatively stable across different stages. Primers used are listed in supplementary Table S7. For microarray analysis, RNA sample quality was first monitored using an Agilent 2100 bioanalyzer. Three microarrays for each genotype were performed at Baylor College of Medicine Microarray Core Facility using the GeneChip™ Mouse Genome 430 2.0 Array (Thermo Fisher Scientific, Waltham, MA). The Bioconductor limma package was used to analyze the microarray data. Briefly, the data was background corrected using the 'normexp' method with an offset of 16 added to the intensities. The background corrected data then was log2-transformed and quantile normalized. Probes without gene description were filtered out before hypothesis testing. Moderated t-statistics were used to test if genes were differentially expressed between the groups of interest and the Benjamini-Hochberg method was used to estimate false discovery rate (FDR). Probes with a *p*-value < 0.01 and a fold change >1.5 were considered the top differentially expressed probes (genes).

### Western blot analysis

Heart lysates were prepared from flash-frozen mouse hearts and run on SDS-PAGE gels. Western blot analysis was conducted following an established procedure 12. Antibodies against Grx3 and GAPDH were used at 1:1000 and 1:2000 dilution, respectively.

### Statistical analysis

All results were shown as means ± SEM. A two-way ANOVA was used to analyze data. Student's *t* test was used to compare the two genotypes. * *p* <0.05, **p<0.01, and *** *p* <0.001 were used as indicators of the level of significance. For microarray data analysis, moderated t-statistics were used to test if genes were differentially expressed between the groups of interest and the Benjamini-Hochberg method was used to estimate false discovery rate (FDR). Probes with a *p*-value < 0.01 and a fold change >1.5 were considered the top differentially expressed probes (genes).

## Results and Discussion

### Grx3 expression in the heart of DIO mice

**Figure 1:**
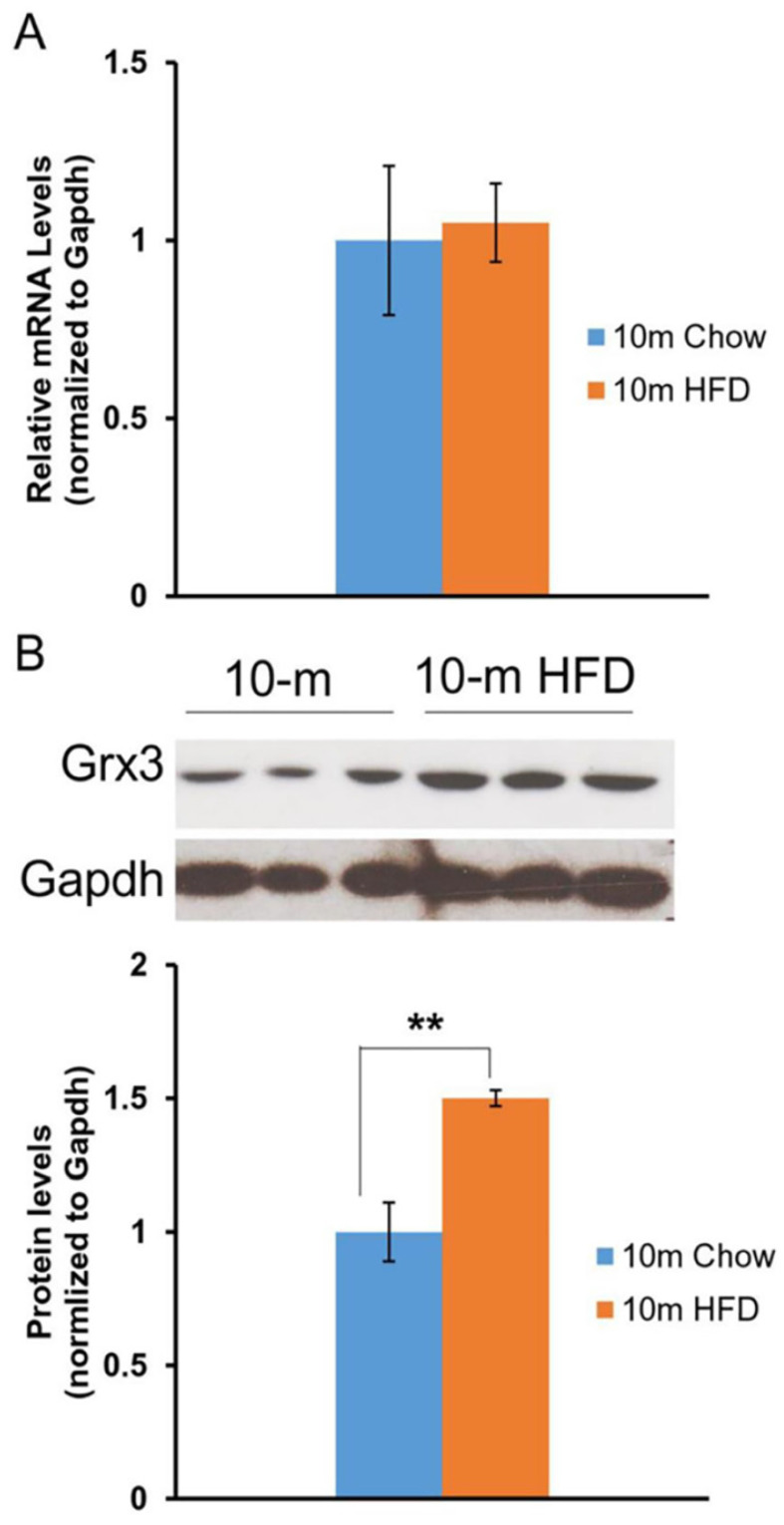
** Grx3 expression in the heart of DIO mice.** (**A**). Q-PCR analysis indicates that *Grx3* mRNA levels were similar in 10-m old mice fed either a chow diet or high fat diet. (**B**). Grx3 protein levels were increased in response to high fat diet feeding. Student *t* test, n=3, * *p*<0.05, ** *p*<0.01.

Our previous studies in mice indicated that *Grx3* is ubiquitously expressed in many tissues and organs 8. Differential expression was observed in the developing mammary glands 14 and also in muscle cells following hydrogen peroxide treatment 8. As regards to the current investigation, *Grx3* expression was also found to decline in the heart during aging 12. Here we wanted to determine the impact of HFD feeding on Grx3 expression in the heart. Both Grx3 transcripts and proteins were measured in the hearts from 10-month-old mice fed either a chow diet- or HFD fed (starting at 2 months of age) by q-PCR and western-blot, respectively (Figure 1). The level of Grx3 transcripts remained unchanged in HFD-fed mice compared to controls (Figure 1A), while the level of Grx3 protein showed a significant increase in response to HFD feeding (Figure 1B). From our previous findings in aged mice, it appears that the HFD doesn't lead to further increase in Grx3 mRNA in 10-month-old mouse hearts as compared to controls but to an increase in the protein level. It is currently unclear what factors are responsible for the discrepancy in Grx3 mRNA and protein levels in the heart under HFD feeding. It has been shown that in mouse skeletal muscle, the high-fat diet feeding significantly down-regulates the muscle extracellular matrix structural gene expression at the mRNA level, but not the protein level 15. The association only seen between the mRNA levels and muscle function could be due to the effect of HFD feeding on muscle structural gene expression (mRNA) and function, rather than structural changes mediating functional changes 15. In our study, it is possible that the HFD feeding potentiates the Grx3 mRNA stability or the turnover of Grx3 proteins in cardiomyocytes. Interestingly, a recent report has shown that AtGRXS17, a homolog of Grx3 from *Arabidopsis thaliana*, is a target of the ubiquitin E3 ligases RGLG3/4 16. Whether Grx3 could be a target of the ubiquitin E3 ligase in the heart and whether turnover of Grx3 could be regulated by HFD feeding warrants future investigation. Nevertheless, the current finding suggests that the increased Grx3 protein levels may provide adaptation to oxidative and metabolic stresses under HFD feeding.

### Enhanced ROS production in the heart of Grx3 CKO mice independently of feeding regime

**Figure 2:**
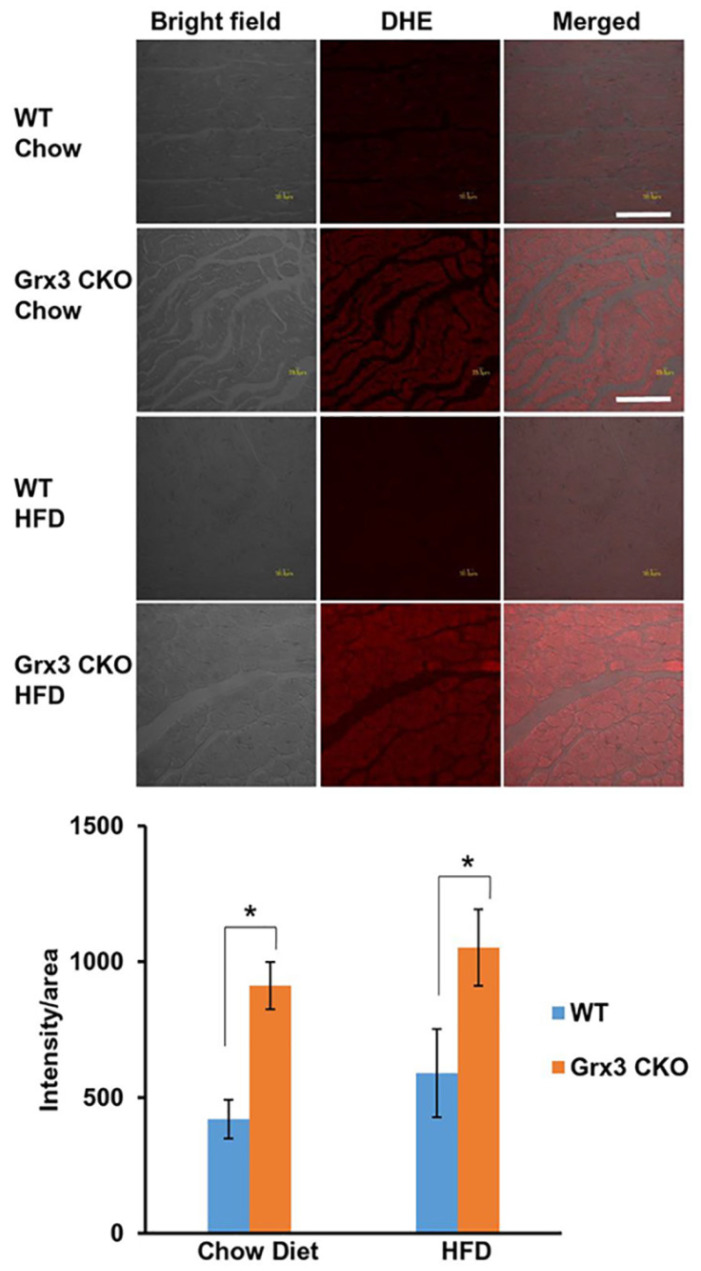
** Grx3 deletion enhanced ROS production in cardiac sections of older Grx3 CKO DIO mice.** (**A**) Representative images showing DHE-fluorescence indicative of elevated ROS levels in cardiac sections of Grx3 CKO and Grx3 CKO DIO mice. Bars= 20µm. (**B**) Quantification of signal intensity per area revealing increased ROS levels in cardiac sections from Grx3 CKO and Grx3 CKO DIO mice compared to littermate controls. * *p*<0.05.

To determine whether the increase of Grx3 protein in the aged mice under HFD provides protection against oxidative stress, we compared loss of Grx3 specifically in the heart (Grx3 cardiac KO (CKO)) against wild type littermate control after being fed HFD or chow diet for 8 months. Histological analysis of left ventricular (LV) sections of 10 months old Grx3 CKO mice and wild type littermate controls fed either a chow diet- or HFD starting at 2 months of age was conducted. Sections were stained with dihydroethidium (DHE) to detect superoxide levels in the LV cardiomyocytes. First, the level of oxidative stress in the heart of 10 months old mice fed with HFD was similar to chow diet-fed mice suggesting that HFD hearts have adapted to the 8-month feeding regime and the previously detected increase in Grx3 protein did not reduce further the ROS level (Figure 2). However, Grx3 CKO hearts from mice fed a chow diet- and HFD exhibited a significant increase in DHE stained signals (Figure 2), indicating increased ROS production in comparison to wild type littermate controls and the essential role of Grx3 in ROS protection (Figure 2). This observation suggests that deletion of Grx3 induces oxidative stress in Grx3 CKO hearts under both chow diet- and HFD-feeding. The NAD^+^/NADH ratio were measured (Figure S1) to further examine whether the redox state were changed in the heart of Grx3 CKO DIO mice compared to wild type controls. Although the levels of ROS were increased in chow-fed Grx3 CKO heart vs wild type controls (Figure 2), the NAD^+^/NADH ratios were similar between the two, suggesting that the redox balance was still maintained under chow-diet feeding. However, under HFD feeding, Grx3 CKO DIO mice showed a significant increase in the NAD^+^/NADH ratio (Figure S1), indicating a redox imbalance in the hearts. There is growing evidence that cardiac energy metabolism and redox balance are crucial for the development and progression of heart diseases including heart failure 17-19. Thus, these results suggest that deletion of Grx3 in the Grx3 CKO DIO hearts leads to oxidative stress and redox imbalance that are detrimental to cardiac function and eventually cause the progression of heart failure in these mice (see below).

### Disruption of Grx3 has impaired cardiac function in DIO mice

To determine if the increase of Grx3 protein in old mice fed with HFD leads to change in metabolic stress, we compared Grx3 CKO mice and the wild type littermate controls following 1 month (young) or 8 months (old) of HFD. Both feeding schedules were started at 2 months of age. In young mice fed HFD, a lack of Grx3 expression in the heart did not affect heart function or growth compared to littermate controls following one month of HFD feeding. No significant difference between Grx3 CKO and controls were measured in body weight, heart weight, heart-to-body weight ratio, and heart rate (Figure S2A-D). In addition, assessment of the LV function by echocardiography revealed no differences in cardiac contractility or dimensions (Figure S2E-H) and there was no difference detected in LV ejection fraction and fractional shortening between CKO mice and littermate controls (Figure S2I-J).

**Figure 3:**
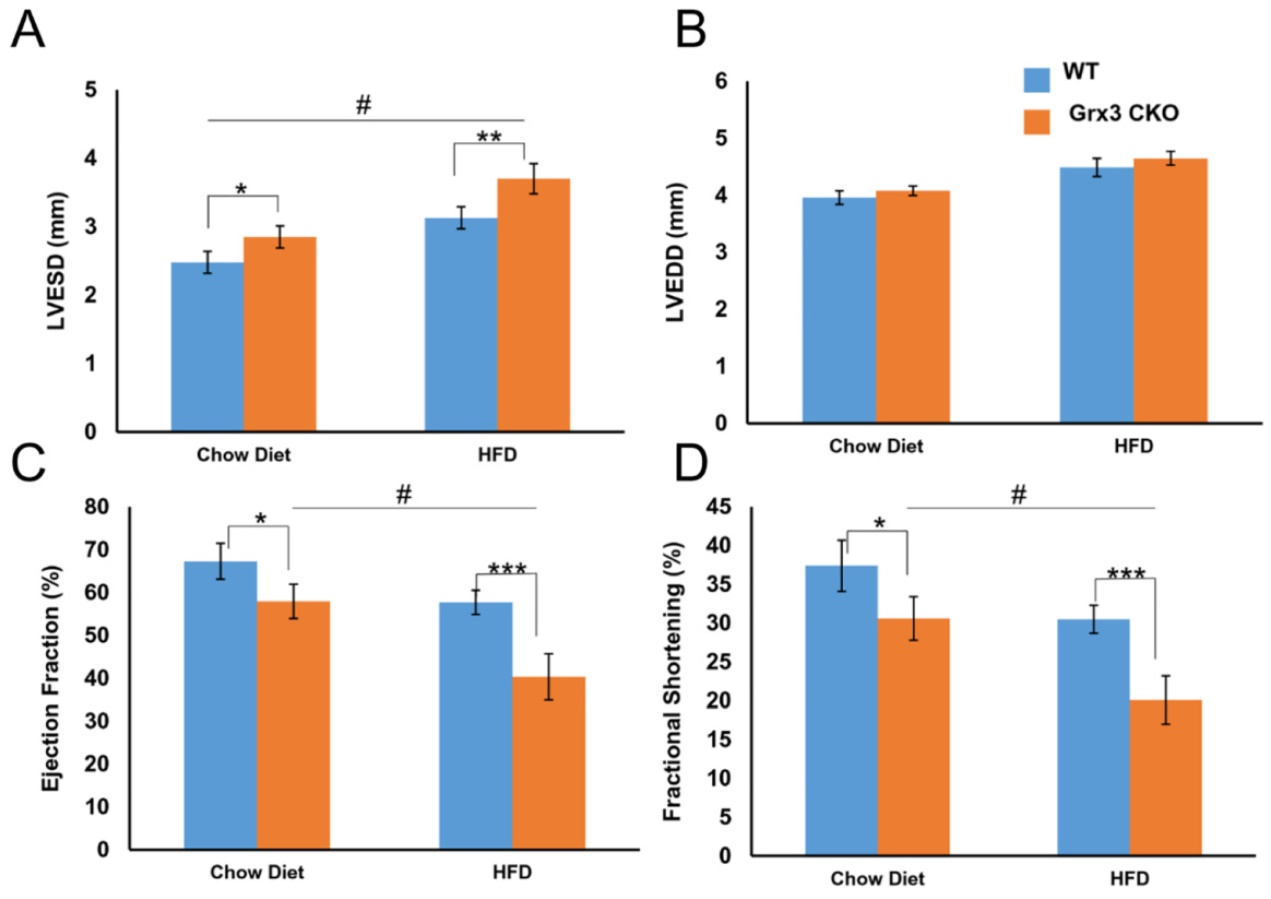
** Cardiac dysfunction and heart failure in Grx3 CKO DIO mice.** *In vivo* echocardiography tracings of 10-m old chow diet- (n=6) and HFD-(n=8) fed controls and Grx3 CKO DIO mice showing left ventricular dilation and impaired contractility. Quantification of echo tracings revealed (**A**-**B**) increased left ventricular end-systolic diameters (LVESD), but not end-diastolic (LVEDD) in Grx3 CKO and Grx3 CKO DIO mice; (**C**-**D**) a significantly decline in ejection fraction (EF) and fraction shortening (FS) in Grx3 CKO and Grx3 CKO DIO mice compared to littermate controls. * *p*<0.5, ** *p*<0.01, and *** *p*<0.001 indicate a significance between Grx3 CKO mice and their littermate controls. # indicates a significance between chow diet- and HFD-fed Grx3 CKO mice.

We also tested the same condition in older mice after being fed HFD for 8 months. Grx3 CKO mice and wild type littermate controls both gained weight and became obese (Figure S3A) compared to chow diet fed mice (WT: 35.1±6.3g vs CKO: 41.5±8.2g; *p*=0.438). As with younger mice, there was little difference between CKO and control DIO mice in body weight (51.1±1.6g vs. 53.2±1.2g; *p*=0.302), heart weight (223.6±9.3mg vs. 226.6±8.4mg; *p*=0.816), heart-to-body weight ratio (4.4±0.2 vs. 4.3±0.1; *p*=0.555) (Figure S3A-C). Interestingly, the heart rate of the CKO DIO mice was significantly higher than that of their littermate DIO controls (553.4±22.1 vs. 461.5±12.3; *p*<0.001) (Figure S3D). The *in vivo* cardiac function analyses using echocardiography revealed a drastic LV dysfunction was observed in Grx3 CKO DIO mice (Figures 3, S3). In detail, LV end-systolic diameter, not end-diastolic diameter was significantly increased in Grx3 CKO DIO hearts (LVESD: 3.7±0.2; LVEDD: 4.7±0.1) compared to littermate DIO controls (LVESD: 3.1±0.2; LVEDD: 4.5±0.2; *p*<0.01, *p*=0.245) (Figure 3A, B). This change was also observed in chow diet fed mice, but with lower diameter (Figure 3A, B). These changes were concomitant with drastic decline in fractional shortening and ejection fraction in Grx3 CKO (EF: 40.3±5.4; FS: 20.1±3.0) versus wild type (EF: 57.7±2.8; FS: 30.5±1.8; *p*<0.001) (Figure 3C, D), along with decreased cardiac output (CO) (21.3±1.7 vs. 24.6±2.1; *p*=0.096) and stroke volume (39.0±3.6 vs. 53.2±4.4; *p*<0.01) (Figure S3H, I). Similar but milder decline in fractional shortening and ejection fraction in Grx3 CKO chow diet fed mice were observed (Figure 3C, D).

Overall, these results indicated that at a young age, high fat diet feeding did not impair cardiac function of Grx3 CKO compared to littermate controls (Figure S2), which is consistent with the our previous findings 12. Grx3 CKO mice started to show some cardiac dysfunction at age of 10 months old even under regular chow diet feeding (Figure 3). However, DIO significantly exacerbated cardiac function in Grx3 CKO mice compared to littermate controls (Figures 3, S3), suggesting that the nutritional imbalance might potentiate cardiac energy metabolism in Grx3 CKO mice.

### Identification of differential expressed genes (DEGs) in Grx3 CKO DIO mice

To gain insight into the transcriptional changes occurring in the heart of old mice lacking Grx3 under HFD, four libraries, two from Grx3 CKO hearts and two from littermate controls fed either chow diet- or HFD (3 biological replicates for each library) were constructed and subjected to DNA microarray analysis. At a 1.5-fold change cut off, a total of 1175 DEGs were identified, of which 178 (127 up- and 51 down-regulated), 796 (409 up- and 387 down-regulated), 87 (64 up- and 23 down-regulated), and 114 (75 up- and 39 down-regulated) were identified between four comparisons (Grx3 CKO vs control hearts under chow diet feeding, Grx3 CKO hearts vs controls under HFD feeding, WT mice under chow diet vs HFD diet feeding, and Grx3 CKO hearts under chow diet vs HFD feeding) (Figure S4). To validate the microarray analysis results, 9 genes having significantly differential expression in Grx3 CKO DIO mice compared to DIO littermate controls were selected to conduct q-RT-PCR analysis. As shown in Figure S5, the results indicated that 8 out of 9 genes having high correlation between the q-RT-PCR and microarray analysis data, which validated the transcriptome results.

**Figure 4:**
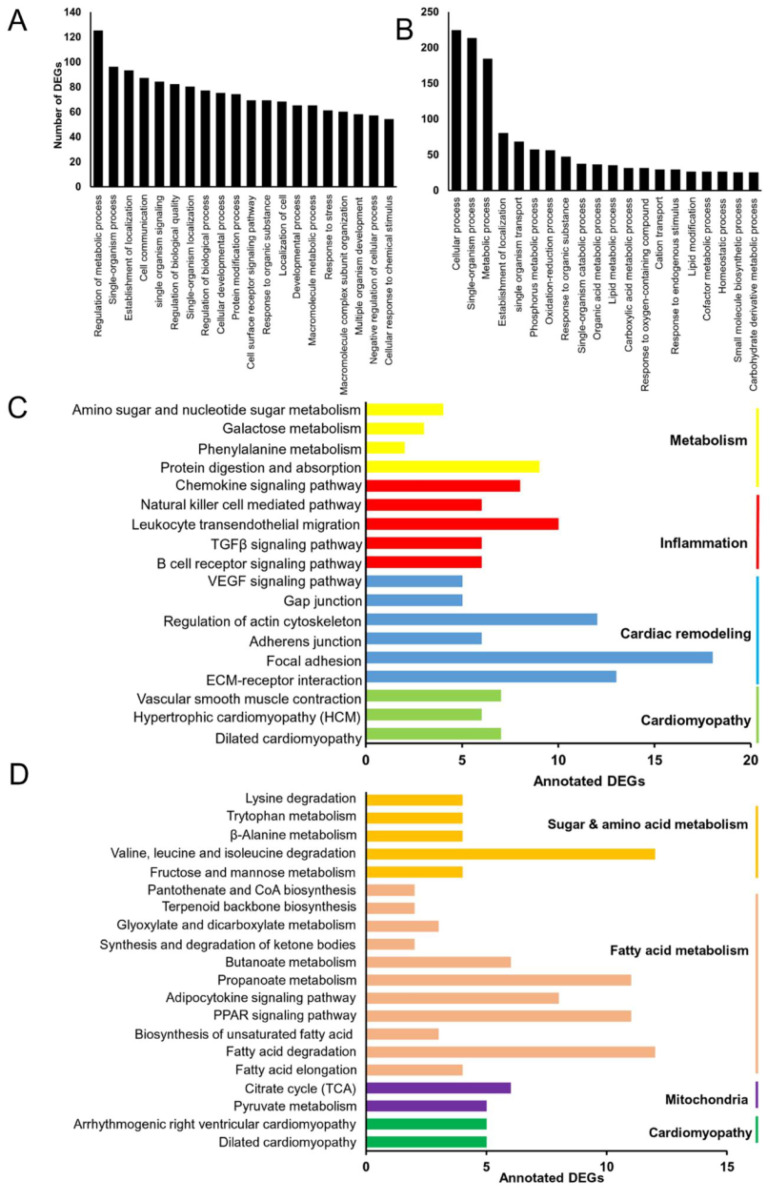
** Identification of differentially expressed genes (DEGs) in Grx3 CKO DIO mice.** (**A**-**B**) GO enrichment of identified up-regulated (**A**) and down-regulated (**B**) DEGs in Grx3 CKO DIO mice compared to littermate DIO controls. (**C**-**D**) KEGG pathway assignment of identified up-regulated (**C**) and down-regulated (**D**) DEGs in Grx3 CKO DIO mice compared to littermate controls.

To further explore the function of the DEGs in Grx3 CKO DIO mice compared to DIO littermate controls (Figure S6, Tables S1, S2), Gene Ontology (GO) enrichment analyses was utilized to group the DEGs according to biological processes (Tables S3, S4). As shown in Figure 4A, the top five represented groups were “regulation of metabolic process”, “single-organism process”, “establishment of localization”, “cell communication”, and “single-organism signaling” for the up-regulated DEGs, while “cellular process”, “single-organism process”, “metabolic process”, “establishment of localization”, and “single-organism transport” were the top five represented groups for the down-regulated DEGs (Figure 4B).

As a step toward assigning each DEG to a specific pathway, Kyoto encyclopedia of genes and genomes (KEGG) analysis was conducted. For the upregulated DEGs, a total of 133 DEGs were assigned into 18 KEGG pathways, which composed four categories: cardiomyopathy, cardiac remodeling, inflammation, and metabolism (Figure 4C). Among the four groups, the inflammation and cardiac remodeling groups have the richest numbers of pathways (Figure 4C) which include the pathway associated with focal adhesion (mmu04510, 18 genes), extracellular matrix (ECM)-receptor interaction (mmu04512, 13 genes), regulation of actin cytoskeleton (mmu04810, 12 genes), TGFβ signaling (mmu04350, 6 genes), and VEGF signaling (mmu04370, 5 genes) (Figure 4C). These results suggest that up-regulation of inflammatory responses and cardiac tissue remodeling may play an important role in developing cardiac dysfunction in Grx3 CKO DIO mice. For the down-regulated DEGs, a total of 83 DEGs were assigned into 19 KEGG pathways which could be grouped into four categories: cardiomyopathy, mitochondria, fatty acid metabolism, and sugar & amino acid metabolism (Figure 4D). In these down-regulated pathways, the ones associated with mitochondria and fatty acid metabolism groups are the most common, which include PPAR signaling (mmu03320, 11 genes), Adipocytokine signaling (mmu04920, 8 genes), propanoate metabolism (mmu00640, 11 genes), and the citrate cycle (TCA cycle, mmu00020, 6 genes) (Figure 4D). These results suggest that down-regulation of mitochondrial function and fatty acid metabolism may contribute to cardiac energy metabolism imbalance and play a crucial role in the pathogenesis of heart failure in Grx3 CKO DIO mice.

### Identification of gene signatures in Grx3 CKO DIO mice

#### Gene clusters involved in cardiomyopathy in Grx3 CKO DIO mice

**Figure 5:**
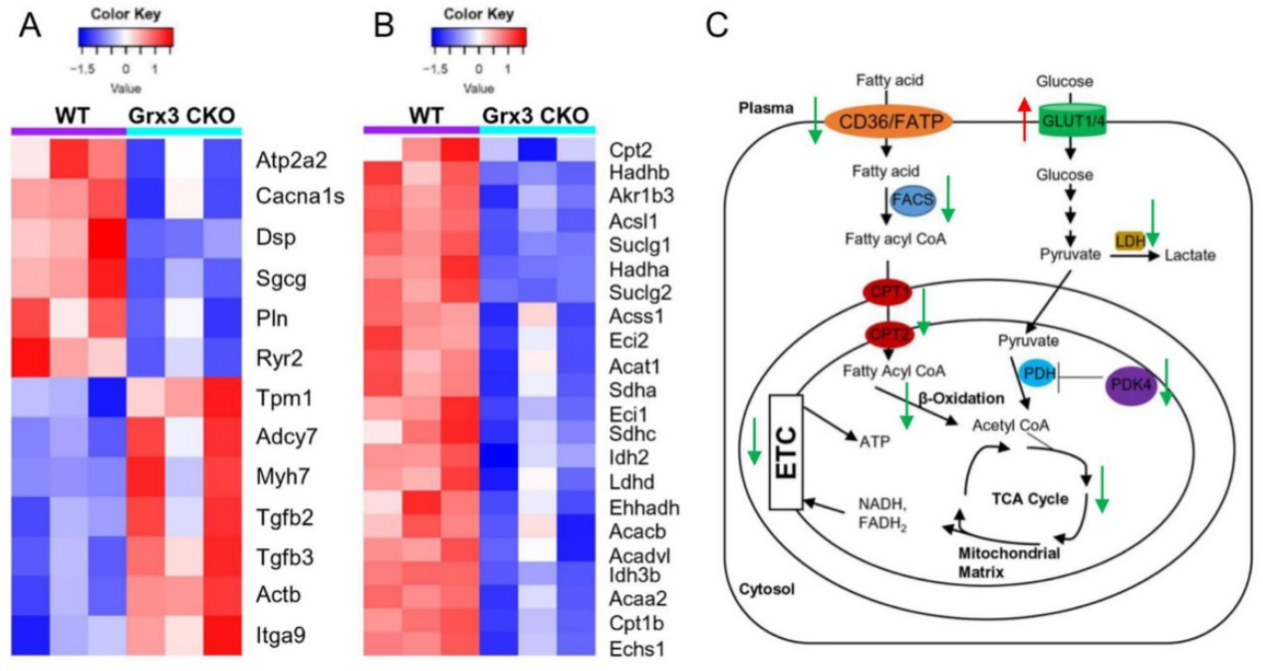
** Identification of gene signatures in Grx3 CKO DIO mice.** (**A**) Heat map diagrams show gene clusters involved in cardiomyopathy in Grx3 CKO DIO mice. (**B**) Heat map diagrams show gene clusters involved in fatty acid metabolism and mitochondrial function. (**C**) Overview of fatty acid and glucose oxidation in cardiomyocytes and alteration of gene expression in Grx3 CKO DIO mice.

Changes in gene expression play an important role in the development of cardiac hypertrophy and heart failure 20-22. According to the KEGG annotations, 13 DEGs involved in dilated cardiomyopathy were identified in Grx3 CKO DIO mice compared to DIO controls (Figures 4C, 4D, 5A). The expression of six genes, an ATPase sarcoplasmic/endoplasmic reticulum Ca^2+^ transporting 2 (*Atp2a2* or *Serca2a*), calcium voltage-gated channel subunit alpha1 S (*Cacna1s*), desmoplakin (*Dsp*), sarcoglycan gamma (*Sgcg*), phospholamban (*Pln*), and ryanodine receptor 2 (*Ryr2*) were significantly reduced in Grx3 CKO DIO mice compared to DIO controls (Figure 5A). All six genes encode proteins that are involved in muscle cell contraction, cardiac calcium transport and calcium handling. Previous studies demonstrated that reduction of *Serca2a*, *Ryr2*, and *Pln* in the heart was significantly correlated with the development of human dilated cardiomyopathy and heart failure 7, 23, 24. DSP is the most abundant desmosomal protein, which binds intermediate filament cytoskeletons to desmosomal plaques and desmosomes are major cellular junctions that are abundant in tissues undergoing constant physical stress, like the heart 25. Mutations in *Dsp* lead to the early onset severe arrhythmias, left ventricular cardiomyopathy, and heart failure 26, 27. Although less is known about the function of *Sgcg* and *Cacna1s* in the pathogenesis of cardiomyopathy, previous reports indicated that disruption of the sarcoglycan complex and/or mutations in the cardiac voltage-dependent L-type calcium channel (LTCC) have been associated with severe cardiomyopathy and a number of inherited cardiac arrhythmia syndromes 28, 29. In addition, *Hrc* (histidine rich calcium binding protein), *Scn4b* (sodium voltage-gated channel beta subunit 4), *Kcnk3* (potassium two pore domain channel subfamily K member 3), *Atp2a1* (*Serca1a*, ATPase sarcoplasmic/endoplasmic reticulum Ca^2+^ transporting 1), *Slc8a3* (solute carrier family 8 member A3) were significantly decreased in Grx3 CKO DIO mice (Figure S7). Overall, these findings imply that down-regulation in the expression of those genes may contribute to dysfunction of cardiac excitation-contraction in Grx3 CKO DIO mice (Figures 3, S3) and alteration of Ca^2+^ signaling and handling in hearts that lack Grx3 expression 7, 12.

In contrast, seven genes encoding tropomyosin 1 (*Tpm1*), adenylate cyclase 7 (*Adcy7*), myosin heavy chain 7 (*Myh7*), actin beta (*Actb*), integrin subunit alpha 9 (*Itga9*), transforming growth factor beta 2 (*Tgfb2*), and transforming growth factor beta 3 (*Tgfb3*) were significantly up-regulated in Grx3 CKO DIO mice compared to DIO controls (Figures. 4C, 4D, 5A). *Tpm1*, *Myh7*, and *Actb* are known to be involved in the pathogenesis of dilated cardiomyopathy 30. Enhanced expression of *Myh7* is a hallmark in the development of hypertrophic cardiomyopathy (HCM) in humans and animal models 31. Mutations and altered expression of *Tpm1* and *Actb* lead to cardiac hypertrophy and heart failure 32-34. *Adcy* encodes adenylate cyclase, an enzyme that can catalyze the conversion of adenosine triphosphate (ATP) into cyclic adenosine monophosphate (cAMP). Increased expression of *Adcy7*, coupled with β-adrenergic receptors and Gαs, may result in enhanced cAMP-mediated signaling which could contribute to the pathogenesis of dilated cardiomyopathy 35. Integrins are cell surface receptors that can potentiate signals from transforming growth factor β (TGFβ) and play essential roles in the fibrotic process 36. Increased expression of *Itga9*, *Tgfb2*, and *Tgfb3* in Grx3 CKO DIO mice may promote myofibroblast proliferation and the fibrotic process that result in cardiomyocyte apoptosis and heart failure 37-39.

#### Gene clusters involved in cardiac tissue remodeling and inflammation

Cardiac remodeling and inflammatory responses are strongly associated with extreme obesity that may lead to the development of heart failure in obese subjects and animal models as well 40, 41. Based on the KEGG enrichment analysis, 60 DEGs were assigned to the category of tissue remodeling and inflammation (Figure 4C, Table S5). Eleven genes, encoding collagen type III alpha 1 chain (*Col3a1*), collagen type V alpha 2 chain (*Col5a2*), collagen type VI alpha 1 chain (*Col6a1*), collagen type I alpha 1 chain (*Col1a1*), collagen type I alpha 2 chain (*Col1a2*), thrombospondin 3 (*Thbs3*), thrombospondin 4 (*Thbs4*), cartilage oligomeric matrix protein (*Comp*), fibronectin 1 (*Fn1*), tenascin C (*Tnc*), and Itag9, were significantly up-regulated in Grx3 CKO DIO mice compared to DIO controls (Figure 4C, Table S5). These genes are involved in ECM interaction and focal adhesion signaling. The ECM is a complex architectural network that play a crucial role in the pathogenesis of heart failure with a reduced ejection fraction 42 as seen in Grx3 CKO DIO mice (Figures 3, S3). The aberrant signal transductions in ECM interaction and focal adhesion have been shown to be crucial for myocardial remodeling and its progression to heart failure 43-48.

Epidermal growth factor receptor (*Egfr*) and fibroblast growth factor receptor 1(*Fgfr1*)-mediated signaling is involved in gap junctions (GJs) and adherens junctions (AJs) and critical for cardiac remodeling 49, 50. For example, cardiac-specific over-expression of epidermal growth factor receptor (*ErbB2*) induced hypertrophic cardiomyopathy 51. Both *Egfr* and *Fgfr1* were found to be up-regulated in Grx3 CKO DIO mice (Table S5). Interestingly, EGFR-mediated signaling, like ErbB2, is down-regulated in Grx3 KD breast cancer cells 13, suggesting that Grx3 may play different roles in heart diseases and cancer.

VEGF and TGFβ signaling pathways are important in inflammation and cardiac remodeling 38, 52, 53. Most significantly, both VEGF and TGF signaling pathways, which include genes encoding protein kinase C beta (*Prkcb*), Rac family small GTPase 2 (*Rac2*), phospholipase A2 group IVA (*Pla2g4a*), prostaglandin-endoperoxide synthase 2 (*Ptgs2*), sphingosine kinase 1 (*Sphk1*), cyclin dependent kinase inhibitor 2B (*Cdkn2b*), *Comp*, *Tgfb2*, *Tgfb3*, *Thbs3*, and *Thbs4*, were activated in Grx3 CKO DIO mice compared to DIO controls (Figure 4C, Table S5), which may contribute to the progression to heart failure in Grx3 CKO DIO mice 38, 54-59.

#### Gene clusters involved in fatty acid metabolism and mitochondrial function

**Figure 6:**
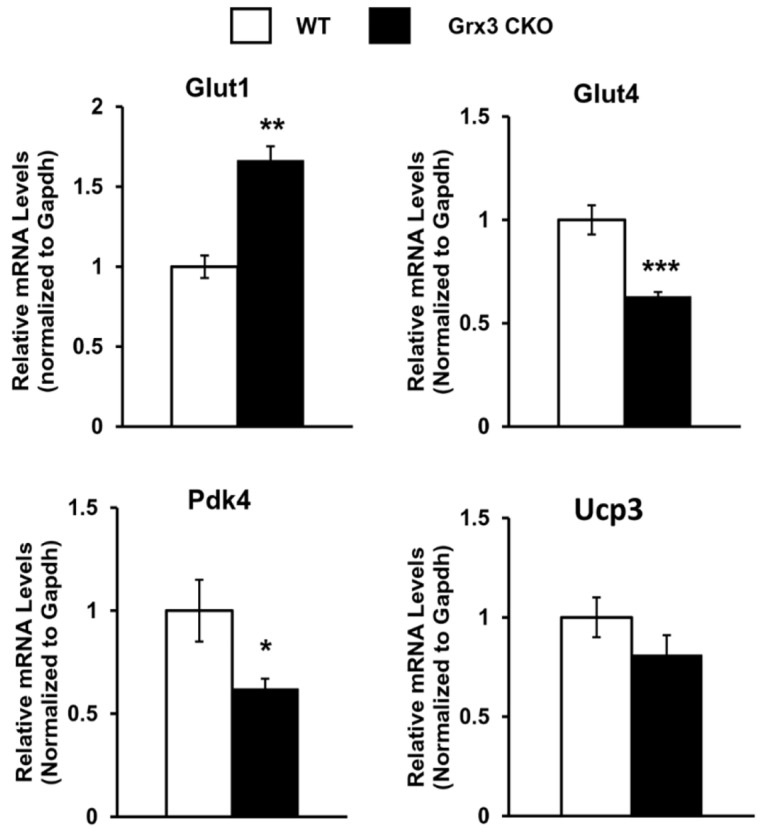
** Gene expression in the Grx3 CKO DIO heart.** Q-PCR analysis indicates an increased *Glut1,* but reduced *Glut4* and *Pdk4* mRNA levels in the Grx3 CKO DIO heart, while *Ucp3* expression was not altered. Gapdh was used as an internal control. Student *t* test, n=3, * *p*<0.05, ** *p*<0.01, and *** *p*<0.001 indicate the significance vs controls.

Proper energy metabolism is essential for cardiac function. In order to adapt to an altered physiological or metabolic environment, the healthy heart has the capacity to select the most efficient substrate for ATP production, however, this metabolic flexibility is diminished in the failing heart 60. In obese subjects, in particular, alterations in cardiac fatty acid metabolism and mitochondrial function may play a causal role in the development and progression of heart failure because of elevated circulating free fatty acids, lipid accumulation (lipotoxicity), and marked shift in fuel utilization 61-63. In Grx3 CKO DIO hearts with profound cardiac dysfunction and heart failure, expression of genes related to fatty acid oxidation (FAO) and mitochondrial function were significantly decreased in comparison to DIO control hearts (Figures 4D, 5B). A total of 35 annotated DEGs were identified and assigned to 10 KEGG pathways which included fatty acid elongation and degradation as well as PPAR and adipocytokine signaling pathways (Figure 4D). *Cd36* encoding the CD36 molecule, a multifunctional immuno-metabolic receptor, and *Slc27a1* encoding the solute carrier family 27 member 1 (FATP1), important in the uptake of long-chain fatty acid in the heart 64-66, were significantly reduced in Grx3 CKO DIO mice compared to DIO controls (Figure 4D, Table S6). This reduction in expression suggests a likely decrease in fatty acid uptake in cardiomyocytes of Grx3 CKO DIO hearts. *Acsl1* encoding acyl-CoA synthetase long chain family member 1, *Cpt1b* encoding carnitine palmitoyltransferase 1B, and *Cpt2* encoding carnitine palmitoyltransferase 2 are rate-limiting enzymes controlling the mitochondrial uptake of long-chain acyl-CoAs for β-oxidation and are indispensable for energy metabolism in the heart 67-69. Down-regulation of *Acsl1*, *Cpt1b*, and *Cpt2* in Grx3 CKO DIO mice (Figure 5B) implies a reduction of mitochondrial fatty acid acyl-CoA uptake. *Acadvl* encodes a very long chain acyl-CoA dehydrogenase and *Echs1* encodes enoyl-CoA hydratase, short chain 1. These enzymes catalyzes the first and second steps, respectively, in mitochondrial fatty acid β-oxidation which is an essential part of cardiac energy metabolism 70, 71. Acetyl-CoA acyltransferase 2 (*Acaa2*), hydroxyacyl-CoA dehydrogenase subunit alpha (*Hadha*), and hydroxyacyl-CoA dehydrogenase subunit beta (*Hadhb*) form a mitochondrial trifunctional protein complex that is critical for fatty acid β-oxidation 72-74. Reduction of *Acadvl* and *Echs1* combined with a reduction of *Acaa2*, *Hadha*, and *Hadhb* suggest a dysregulation of mitochondrial fatty acid oxidation in Grx3 CKO DIO hearts. In addition, succinate-CoA ligase GDP/ADP-forming subunit α/β (*Suclg1/2*), succinate dehydrogenase complex subunit C (*Sdhc*), succinate dehydrogenase complex flavorprotein subunit A (*Sdha*), isocitrate dehydrogenase (NAD^+^) 3 non-catalytic subunit beta (*Idh3b*), and isocitrate dehydrogenase (NADP^+^) 2 (*Idh2*) are key enzymes involved in the citrate cycle (TCA) which is essential for mitochondrial bioenergetics in cardiomyocytes 75. Decreased expression of these genes (*Suclg1/2*, *Sdha*, *Sdhc*, *Idh3b*, and *Idh2*) suggested insufficient production of ATP from the mitochondrial TCA in Grx3 CKO DIO hearts (Figure 5B, Table S6). This lack of ATP may be a critical factor in the progression of heart failure 75-79. Most interestingly, peroxisome proliferator-activated receptors (PPARs), the retinoid X receptors (RXRs), and the PPAR co-activator g (PGC-1a) play an essential role in the transcriptional regulation of genes encoding enzymes involved in cardiac fatty acid metabolism 80, 81. In Grx3 CKO DIO hearts, the PPAR signaling pathway (*Pparα*, *Rxrγ*, *Ppargc1α*), especially PPARα targets in the cellular fatty acid oxidation pathway, was significantly down-regulated compared to DIO controls (Figures 4D, 5B, Table S6). Interestingly, while the fatty acid utilization as substrates was diminished in Grx3 CKO DIO mice, glucose uptake and utilization as fuel substrates was enhanced in Grx3 CKO DIO hearts compared to DIO controls as evident by increased expression of *Glut1* and reduced expression of *Pkd4* (Figure 6). Together, these findings suggest that dysregulation of fatty acid oxidation and altered glucose metabolism in cardiomyocytes, a fuel utilization switch, may contribute to cardiac function and heart failure in Grx3 CKO DIO mice 61, 82, 83.

Grx3 is an iron-sulfur binding protein shown to regulate cellular iron homeostasis 84, 85. Deletion of Grx3 results in iron accumulation in the cell, which could generate cellular ROS and cause redox imbalance through the Fenton chemical reaction. Grx3 has been shown to play an important role in cytosolic iron-sulfur assembly transferring [2Fe-2S] clusters to monomeric apo proteins 86-88. Interestingly, a recent study reports that human Grx3 can bind and effectively deliver a [4Fe-4S] cluster to apo iron regulatory protein 1 (IRP1) and convert IRP1 into an aconitase 89. Therefore, these studies suggest that the absence of Grx3 could impair Fe-S cluster assembly, which is required for many mitochondrial metabolic enzymes and nuclear proteins and may increase mitochondrial ROS production in Grx3 CKO DIO hearts. Our current findings indicate that expression of gene clusters involved in mitochondrial energy metabolism was significantly decreased (Figure 5B, Table S6), supporting the notion that mitochondrial function might be impaired in Grx3 CKO DIO heart. However, the mechanisms by which Grx3 regulates ROS and redox homeostasis in cardiomyocytes, especially under HFD feeding, remain to be fully investigated and warrant future study.

In conclusion, disruption of Grx3 increases ROS accumulation in cardiomyocytes and impairs cardiac function leading to heart failure under diet-induced obesity. Transcriptional analysis revealed a significant decrease in expression of genes related to fatty acid uptake, mitochondrial transfer, beta-oxidation, TCA, and PPAR signaling pathway and an increase in expression of genes related to glucose uptake and utilization, suggesting a metabolic switch occurred in Grx3 CKO DIO hearts (Figure 5C). Our findings indicate that Grx3 plays a critical role in cardiac energy metabolism and offers a clue to the elaborate regulatory interplay between redox homeostasis and metabolic control in the heart.

## Supplementary Material

Supplementary figures.

Supplementary table 1.

Supplementary table 2.

Supplementary table 3.

Supplementary table 4.

Supplementary table 5.

Supplementary table 6.

Supplementary table 7.
