# Supplementary material for: Crucial Role of Mammalian Glutaredoxin 3 in Cardiac Energy Metabolism in Diet-induced Obese Mice Revealed by Transcriptome Analysis
Source: Int J Biol Sci. 2021 Jul 13;17(11):2871–83. doi: 10.7150/ijbs.60263 (PMC8326124; doi:10.7150/ijbs.60263)
Supplement: Supplementary file 1 — Supplementary figures. [file ijbsv17p2871s1.pdf]

Supplementary Figure S1

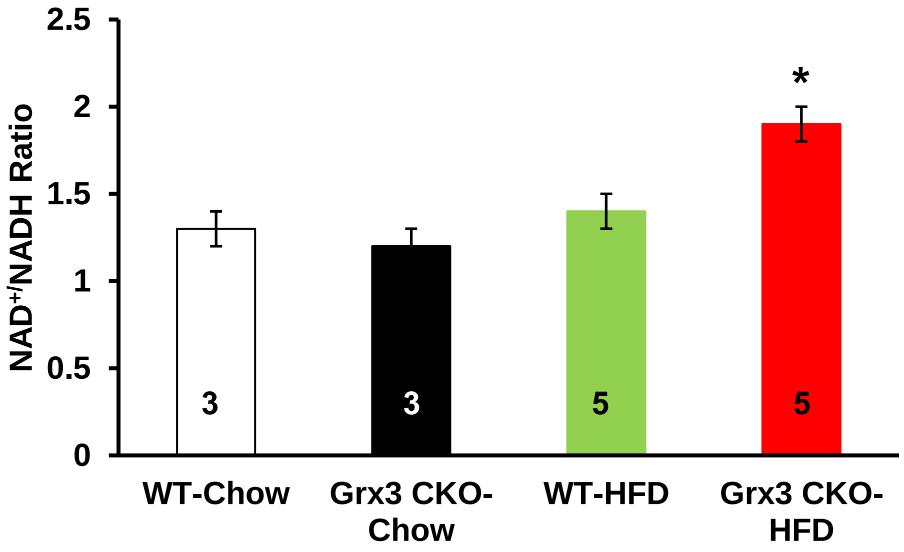

Supplementary Figure S1. Cardiac NAD^+/NADH ratio in Grx3 CKO DIO mice. The levels of NAD^+ and NADH and the calculation of the NAD^+/NADH ratio in chow- and HFD-fed Grx3 CKO DIO mice and littermate controls were determined. n=3 or 5, * $p<0.05$ indicates a significant difference between HFD-fed Grx3 CKO mice vs controls.

Supplementary Figure S2

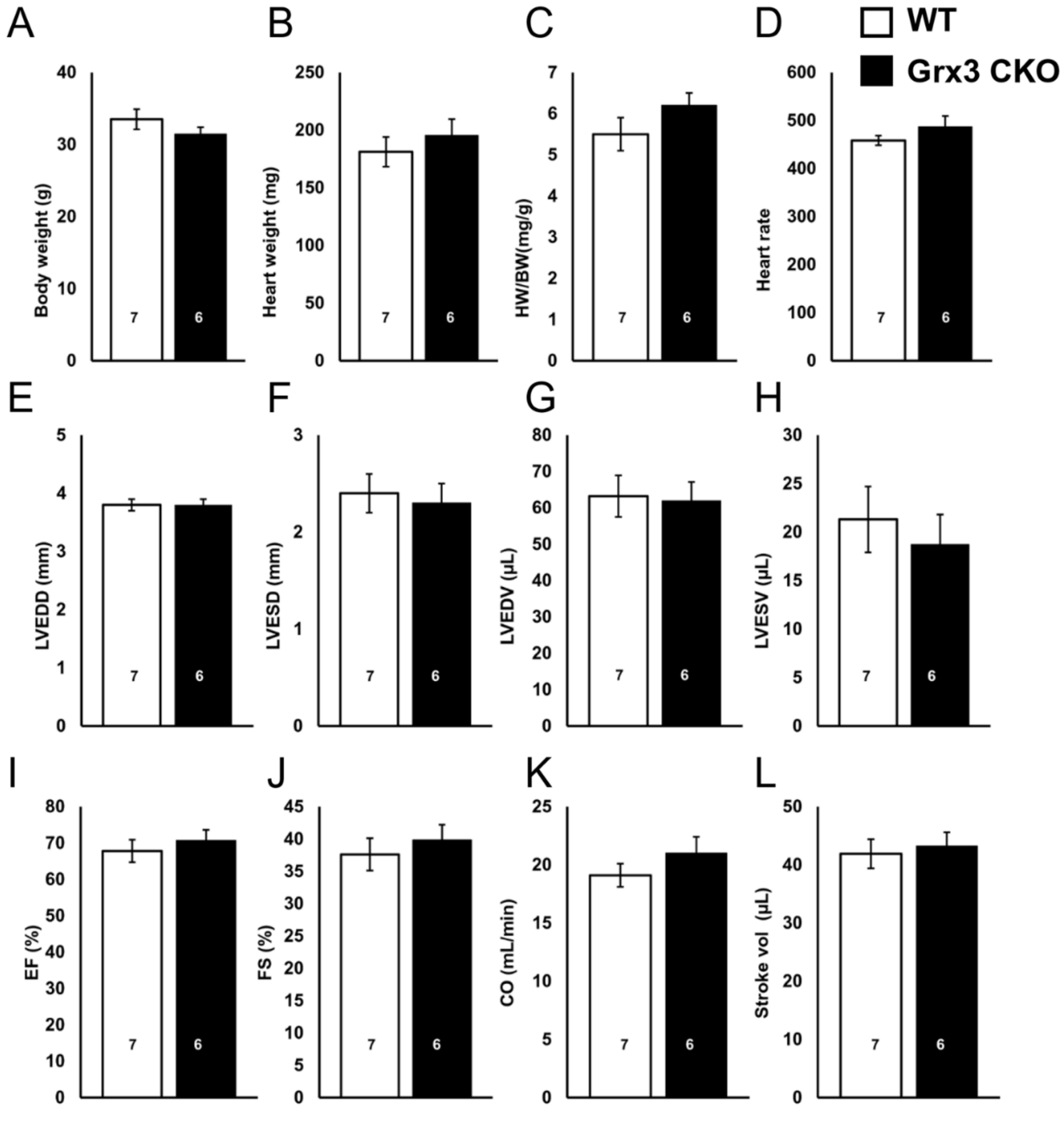

Supplementary Figure S2. Assessment of LV function by echocardiography in young Grx3 CKO mice. Body weight **(A)**, Heart weight **(B)**, HW to BW ratio **(C)**, and Heart rate **(D)** between Grx3 CKO and littermate controls. **(E-L)** Quantification of echo tracings revealed no differences in cardiac contractility or dimensions. Furthermore, there was no difference in LV ejection fraction and fractional shortening in HFD-fed 3-m old Grx3 CKO mice and littermate controls.

Supplementary Figure S3

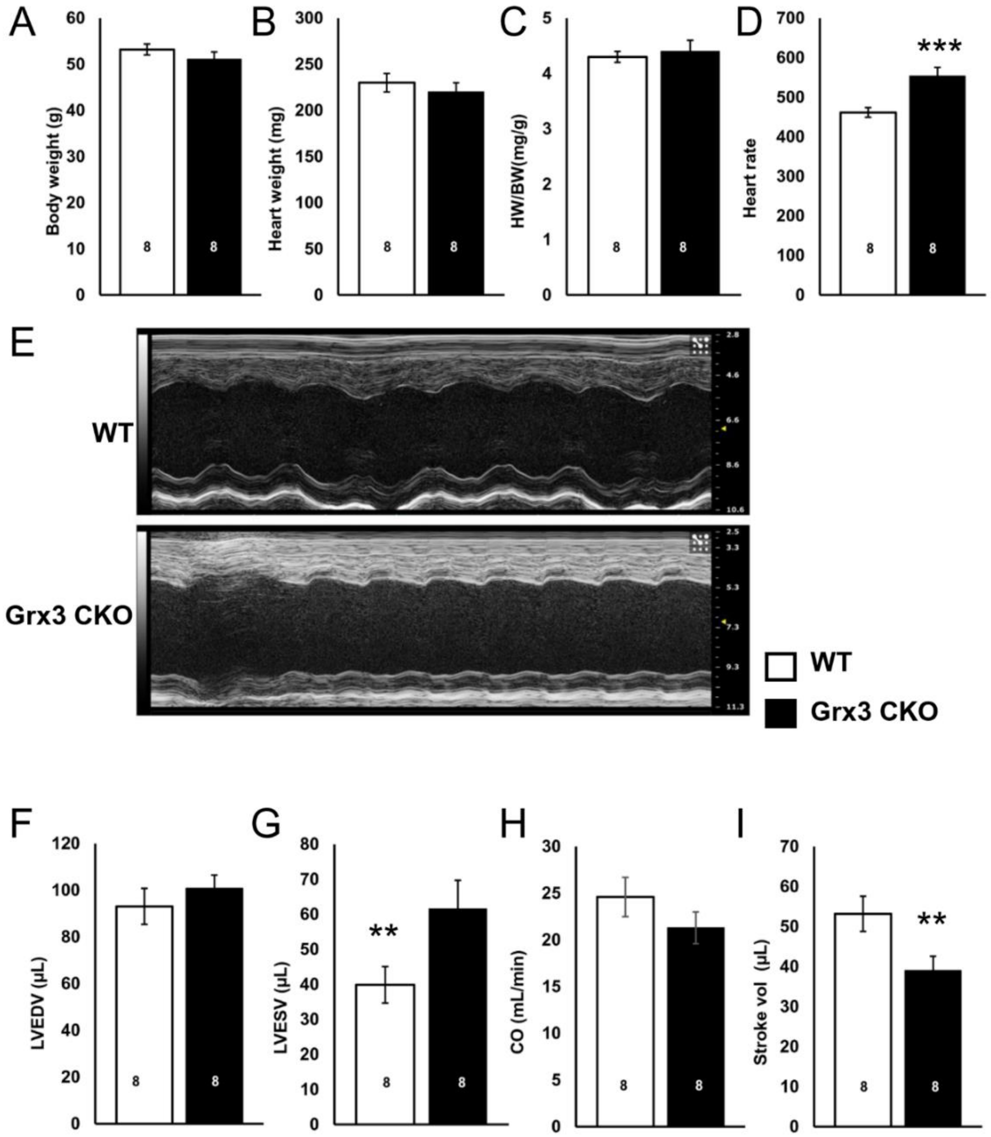

Supplementary Figure S3. Cardiac dysfunction and heart failure in Grx3 CKO DIO mice. Body weight **(A)**, Heart weight **(B)**, HW to BW ratio **(C)**, and Heart rate **(D)** between Grx3 CKO DIO and littermate DIO controls (*** $p<0.001$). **(E)** Representative M-mode echocardiography tracings of 10-m old HFD fed control and Grx3 CKO DIO mice showing left ventricular dilation and impaired contractility. **(F-I)** Quantification of echo tracings revealed **(F-G)** increased left ventricular end-systolic volume (LVESV), but not end-diastolic volume (LVEDV), **(H-I)** reduced cardiac output (CO) and stroke volume (SV). (** $p<0.01$, *** $p<0.001$).

Supplementary Figure S4

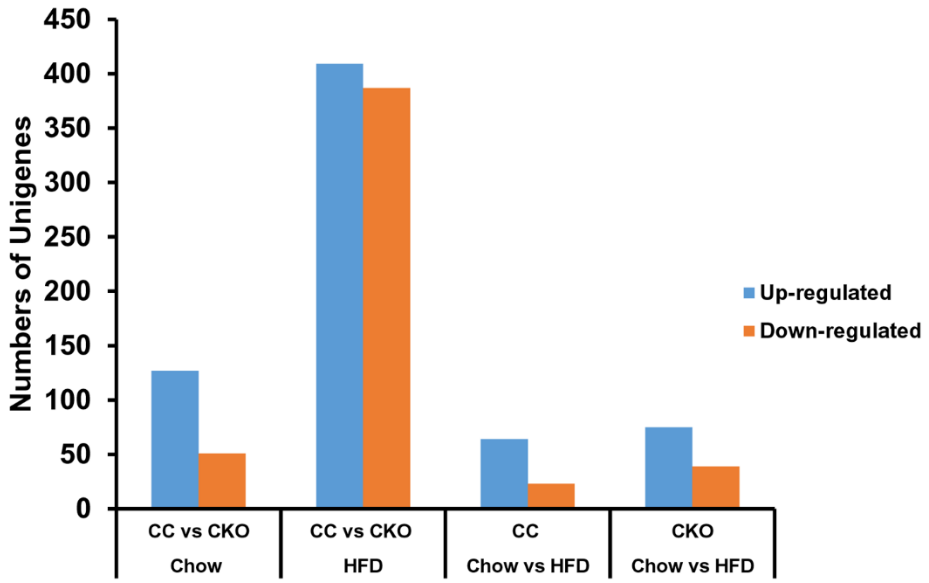

Supplementary Figure S4. Numbers of up-regulated and down-regulated DEGs in different comparison groups.

Supplementary Figure S5

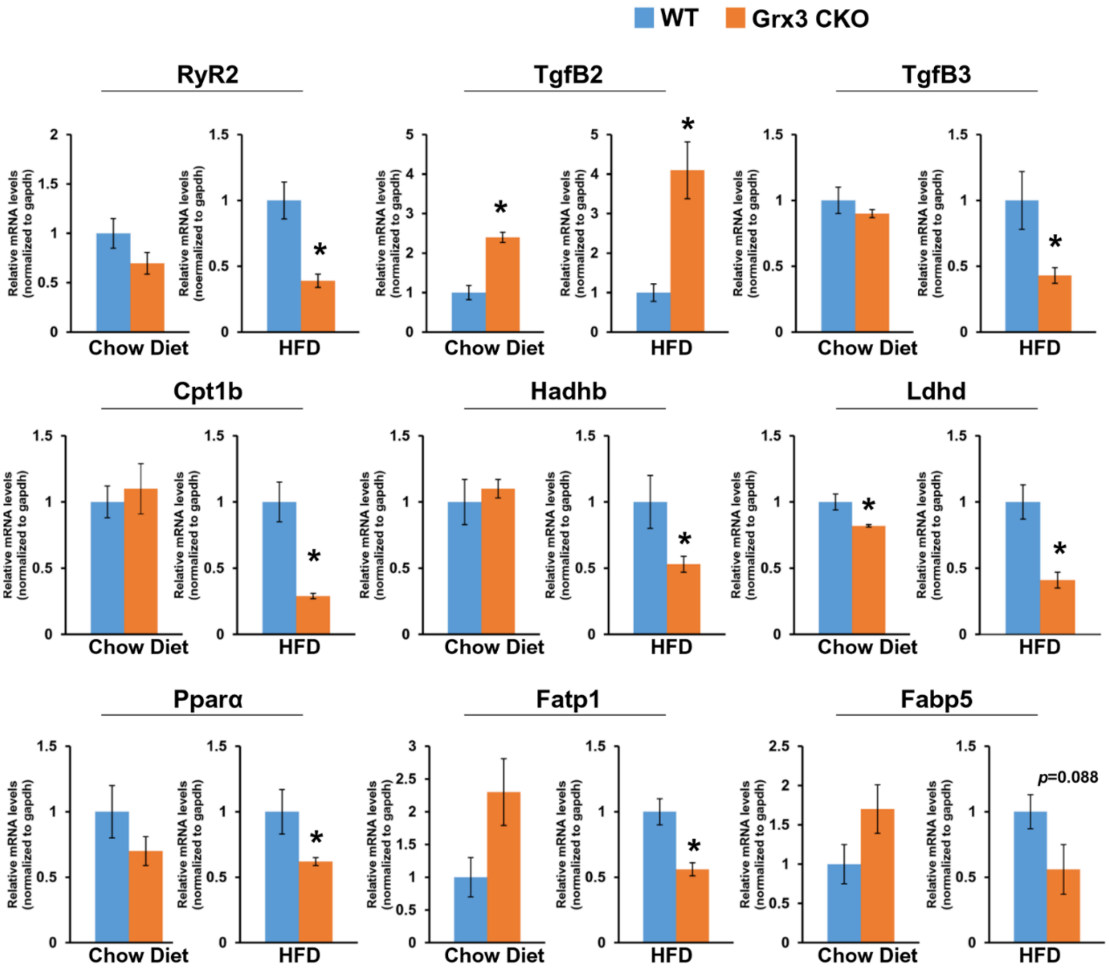

Supplementary Figure S5. Gene expression in the Grx3 CKO DIO heart. Q-PCR analysis indicates a reduced expression levels of genes involved in fatty acid metabolism pathway in the Grx3 CKO DIO heart and an increase in expression of genes involved in cardiac inflammation. Gapdh was used as an internal control. Student *t* test, n=3, * p<0.05 indicates the significance vs controls.

Supplementary Figure S6

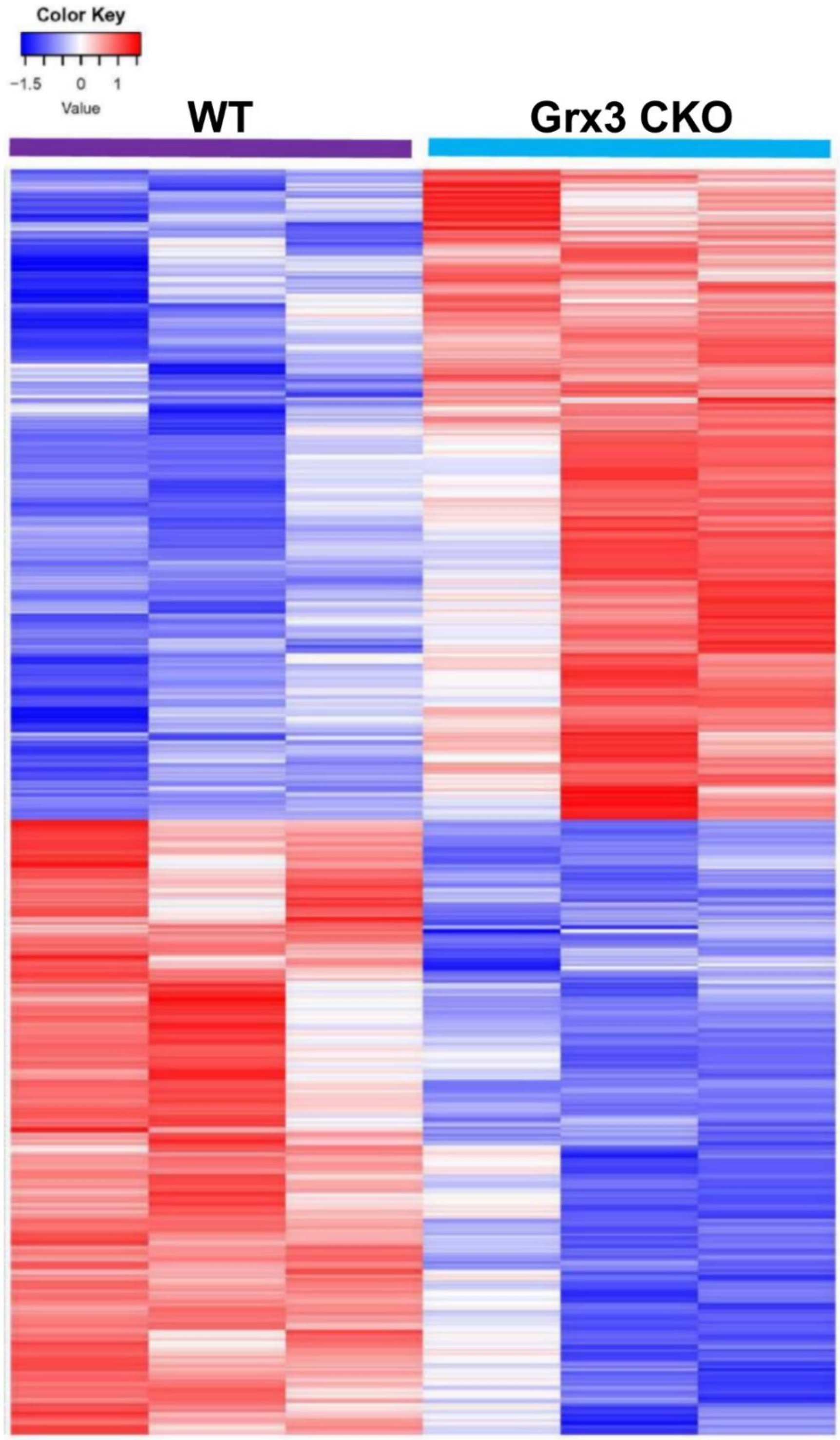

Supplementary Figure S6. Identification of differentially expressed genes (DEGs) in Grx3 CKO DIO mice. Heat map diagrams show DEGs in Grx3 CKO DIO mice compared to littermate DIO controls. $p<0.01$

Supplementary Figure S7

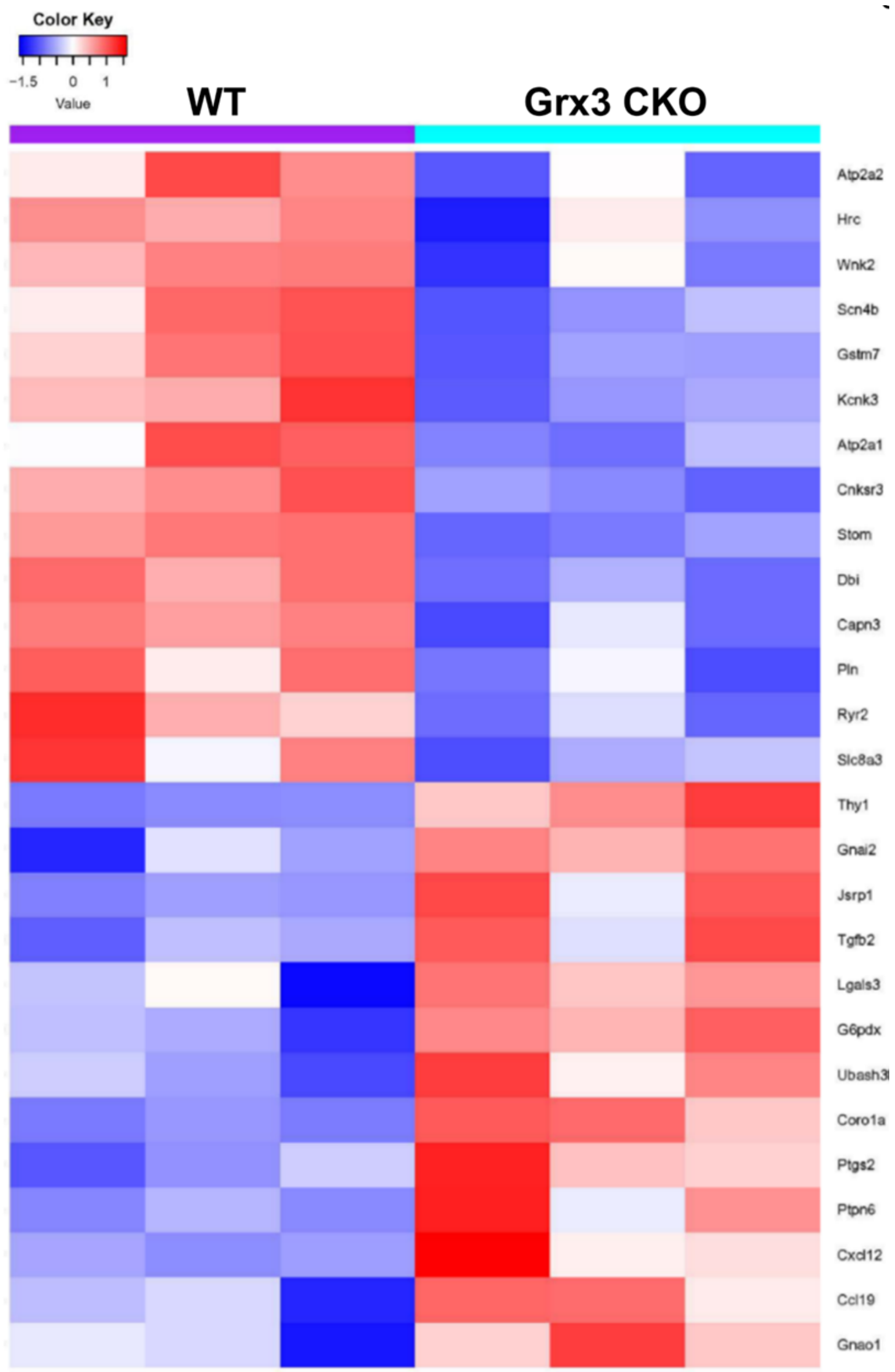

Supplementary Figure S7. Heat map diagrams show gene clusters involved in calcium transport and handling in Grx3 CKO DIO mice. ***p*****<0.01**
